# Preferences for Income Redistribution in Unequal Contexts: Changes in Latin America Between 2008 and 2018

**DOI:** 10.3389/fsoc.2022.806458

**Published:** 2022-05-04

**Authors:** Gonzalo Franetovic, Juan-Carlos Castillo

**Affiliations:** ^1^Department of Social and Political Sciences, University of Milan, Milan, Italy; ^2^Department of Sociology, Universidad de Chile, Santiago, Chile

**Keywords:** redistributive preferences, income, inequality, economic development, Latin America

## Abstract

In a developing and highly unequal region like Latin America, it is crucial to understand the determinants that affect people's support for redistribution of resources from the state. A series of theories focused on self-interest have continuously established a negative link between people's income and their support for the reduction of inequalities through redistribution. Despite this, the evidence is scarce and sometimes contradictory while its study in Latin America is almost non-existent. Using data from the LAPOP Survey between 2008 and 2018, a longitudinal dimension is considered for the first time in the measurement of Latin American redistributive preferences, using hybrid multilevel regression models. In contrast to the evidence from studies conducted in other regions, the results reveal that in Latin America it is not possible to detect a clear association between income and redistributive preferences at specific times, but it is possible when changes occur in countries' levels of inequality and economic development. Likewise, other elements that consistently affect preferences are evident, such as educational level, political ideology, and confidence in the political system. In light of this evidence, comparisons are made with previous research findings in industrialized countries, challenging rationalist theories of justice and solidarity.

## 1. Introduction

The redistribution of resources within a society constitutes one of the basic elements of the social contract and plays a key role in reducing poverty and inequality (Hoffman and Centeno, 2003). Traditional perspectives on redistribution assume that in contexts of high inequality there will be a greater demand for the redistributive action of the state, particularly through the election of representatives who favor redistribution through the political action of governments (assuming a democratic context). For this reason, identifying the degree of people's support for redistribution and understanding the main determinants that explain it is an exercise of great importance, even more so in contexts of high poverty and inequality such as Latin America. Although this region has shown signs of decreasing poverty and inequality (Lustig et al., 2013; Dayton-Johnson, 2015), a large body of evidence concludes that Latin America is the most unequal region in the world (Bértola et al., 2009; Williamson, 2015; CEPAL, 2016) and, more seriously, that it has maintained this position steadily since the middle of the last century (Mann and Riley, 2007). This raises a question about the degree of support for redistribution in highly unequal contexts and its possible role in reducing inequality.

Within this framework, this study is guided by the following question: What do redistributive preferences look like, and how do they change in less industrialized societies with high economic inequality? While it is generally assumed that people with higher incomes will be more resistant to the redistributive action of the state for reasons of self-interest, most research to date has been implemented in comparatively more egalitarian contexts. This situation opens up the question of whether inequality would be an element that would increase pressure for redistribution and thus lessen the differences between individuals of different socioeconomic levels in their redistributive preferences (Dimick et al., 2016, 2018). On the other hand, most research on preferences analyzes this phenomenon in a static way without considering whether changes in inequality levels have an impact on greater or lesser support for redistributive policies.

The lack of studies on redistributive preferences in unequal contexts, and on their change, is mainly due to the scarcity of specific data containing these variables at different moments in time and for a set of countries. Fortunately, the Latin American Public Opinion Project (LAPOP) survey offers for the first time the opportunity to analyze the redistributive preferences in 17 Latin American countries over a 10-year time horizon (2008–2018). Along with the availability of these data, advances in the analysis and modeling of longitudinal change with cross-sectional data (Schmidt-Catran and Fairbrother, 2016) (or more generically, multilevel hybrid models) have also been recently published. So far these models have not been applied to the Latin American region, or even internationally on the topic of preferences, thus representing a double contribution.

This article is structured into five sections. First, evidence regarding individual and contextual determinants in redistributive preferences is discussed, focusing on the self-interest approach and its criticisms. In the second section, the methodology used is described, including details regarding the sample, the variables, and the hybrid multilevel regression models used. Thirdly, the results are presented, divided into two sub-sections: descriptive analysis, identifying national and temporal trends in support for redistribution; and multilevel estimation, presenting the results of the statistical models with emphasis on the analysis of change over time. The fourth section discusses the results in comparison to the literature, and the last section gives an account of the main conclusions that arise from this research as well as its limitations and future lines of study based on the findings.

## 2. Redistribution and Inequality

With the increase in inequalities, the rise in the concentration of wealth, and the crisis of the welfare states across a wide range of countries, preferences for redistribution have become a topic of increasing academic interest (Rueda and Stegmueller, 2019). The study of preferences is inserted in a discussion where it shares ground with attitudes toward the welfare state (Reeskens and van Oorschot, 2012; Roosma et al., 2014; Eger and Breznau, 2017), forms of social solidarity (Janmaat and Braun, 2009), agreement with social policies (Kwon and Curran, 2016), and perception and legitimization of inequalities, among others. Since this study arises from the premise that attitudes toward public policies can be understood by explanations at different levels (Alesina and Giuliano, 2009), the literature review will be structured in two sections: first, regarding factors of an individual nature (Alesina and Giuliano, 2009; Franko et al., 2013; McCall, 2013) and secondly, those at the country level (Edlund, 1999; Kenworthy and McCall, 2007; Isaksson and Lindskog, 2009).

### 2.1. Individual Factors of Redistributive Preferences

#### 2.1.1. Self-Interest, Income, and Objective Position

If there is one certainty within the study of redistributive preferences, it is that the great majority give the theory of the median voter a pioneering and fundamental role (Lübker, 2004; Alesina and Giuliano, 2009; Castillo and Sáez Lozano, 2010; Dhami and Al-Nowaihi, 2010; Keller et al., 2010; McCall, 2013; Berens, 2015b; Castillo et al., 2015). In their classic model, Meltzer and Richard (1981), based on Romer (1975), established that the greater the inequality in countries, the greater the tendency for voters to support social spending, resulting in an increase in the effective redistribution of wealth between rich and poor. This would occur because in more unequal contexts the median-income voter will be poorer than the average-income voter, so most individuals will have incentives to vote for redistribution. In a democratic context of open elections, this would translate into a greater effective redistribution of resources within a society, maintaining a kind of distributive self-regulation.

The median-voter hypothesis is based on the so-called “self-interest approach” perspective that assumes a direct relationship between the socioeconomic position of the subject within the social structure and its interpretations and provisions in terms of distributive justice. It is argued, then, that the position held by the subjects determines a different exposure to the risk of falling into an economically undesirable situation and that the latter would be responsible for generating different patterns of self-interest (Wegener and Liebig, 2000). Thus, this perspective guarantees that the relative position before risk, experienced differently by the subjects, would be an essential condition of the importance attributed to redistribution (Rehm et al., 2012; Barth et al., 2015).

Previous studies have identified a number of factors that are linked to different redistributive preferences. Determinants such as status—in terms of educational or occupational level—or social class of belonging—at the level of position in the productive structure—are used as an expression of self-interest, as well as the labor condition (Gijsberts, 2002). The most commonly analyzed determinant, however, is income. In addition to Meltzer and Richard (1981), Franko et al. (2013) state that belonging to a low-income stratum is consistently associated with greater tendencies to support an increase in redistribution, which would translate into an increase in the tax burden on the richest. This negative relationship between income and redistribution has also been evidenced by Bernasconi (2006), Iversen (2005), Jaeger (2006), and Finseraas (2009), all of whom endorse the significant downward trend in support for redistribution as people's income increases. The explanation for this widely studied relationship is supported by what Szirmai (1986) understands as “absolute deprivation”: people with higher income levels will legitimize greater inequality because a narrowing of the gaps will tend to disadvantage them. Similarly, people with low incomes will prefer less inequality insofar as they will benefit from their current condition.

#### 2.1.2. Homo-Sociologicus and the Critique of Self-Interest

In spite of the support that the theory of self-interest finds in common sense and in a series of investigations, there are also proposals and evidence that distance themselves from the mere instrumental reasons of homo-economicus, pointing out as a counterpart a homo-sociologicus that contemplates culture, values, and beliefs that go beyond personal interest (Etzioni, 1988; Feldman and Zaller, 1992). Therefore, issues such as political identification (Castillo et al., 2013), and trust in the tax system (Alm and Torgler, 2006), as well as religion (Scheve and Stasavage, 2006) are elements that have tended to be related to the configuration of support by redistribution. The same is true for trust in the political system: it is assumed that as long as people consider that government institutions operate based on principles such as efficiency and probity, they are more likely to support welfare policies (Kumlin, 2004), such as redistribution of resources and others.

With respect to Latin America, the action of self-interest in shaping preferences for redistribution has also been questioned. Berens (2015a) has focused his analysis on the characteristics of the region and the differences between formal and informal workers. According to the self-interest approach, people with irregular employment would tend to have a greater support for redistribution while their economic activity, being outside the formal labor system, does not entail the application of associated taxes Schmidt-Catran (2016). However, Berens (2015a) reveals that this relationship would operate in reverse, the interest being more influential on formal than informal workers within the region. However, given that the issue of redistributive preferences has scarcely been studied in the region, our initial approach explores the rather traditional perspective of self-interest, from which the first hypothesis of the study emerges:

*H1: The higher the income level, the less support there is for redistributive policies*.

### 2.2. Contextual Factors of Redistributive Preferences

In addition to the characteristics that define subjects at the individual level, it has been observed that preferences and attitudes in matters of distribution are highly influenced by elements of the context in which these people live (Wegener and Liebig, 1995; Forsé and Parodi, 2007). Given its particular importance in terms of narrowing the economic gaps among the population, we will discuss two major determinants at the national level: inequality and economic development.

#### 2.2.1. Economic Inequality

As we noted earlier for individual factors, according to Meltzer and Richard (1981) the greater the inequality within countries, the greater the likelihood that individuals will agree with redistribution. This relationship can also be considered in a dynamic sense and therefore should apply both “between” countries and “within” countries over time as any increase in inequality in a country will also produce a shift in the average voter-to-median voter ratio, making even greater demand for redistribution across the population foreseeable.

However, the empirical evidence shows that this relationship is more complex than it seems, being non-existent in some cases (Lübker, 2007) and in many others showing even greater tolerance for inequality in societies (Castillo, 2010; Sachweh and Olafsdottir, 2010; Schröder, 2017; Mijs, 2019). Thus, a good number of studies have tended to problematize the applicability of the median-voter theory from a transversal (“between” countries) approach (Alesina and Glaeser, 2004; Kenworthy and Pontusson, 2005) as well as a longitudinal (“within” countries) approach. For example, using data for eight nations between 1980 and 1990, Kenworthy and McCall (2007) conclude that variations in inequality within countries would not be associated with a consequent change in the generosity of redistributive policies. The same is suggested by Schmidt-Catran (2016) in European countries, who finds support for the median-voter theory at the cross-sectional but not at the longitudinal level. Furthermore, contemporary authors have seen how income inequality (Huber and Stephens, 2001; Atkinson et al., 2011), the divisions between included and excluded (Rueda, 2008), and unemployment (Rehm, 2011) have become much more frequent phenomena, without a conducive reduction of inequalities in developed countries. Despite this, Latin American countries seem to show a different trend in recent years where they have seen an expansion of their social policies in favor of the poorest (Mares and Carnes, 2009; Garay, 2010). All of this anticipates possible shortcomings of the classic model of the middle-class voter at the macro level for the Latin American case; however, again arguing from a more traditional and rational perspective, our second hypothesis proposes that:

*H2a (between): Countries' levels of economic inequality will be positively related to support for redistribution*.
*H2b (in): Increases in economic inequality in countries will be positively related to support for redistribution*.

Along with the direct effect of inequality on preferences for redistribution, it is possible to think that the economic inequality of countries could also have a moderating effect, affecting the way in which various individual characteristics are related to the demand for redistribution. Authors such as Lupu and Pontusson (2011) and Luttig (2013), argue that the structure of inequality is particularly relevant. For them, in more unequal societies there would be less difference in redistributive preferences along the different income strata, due to the constitution of a smaller group of privileged people and the consequent emergence of a parochial altruism: feelings of solidarity and affinity mostly shared along the non-benefited population (Fowler and Kam, 2007).

More recently, Dimick et al. (2016, 2018) have strengthened this theoretical field by developing a theory known as “income-dependent altruism.” From this perspective, which combines the approaches of self-interest and altruism (Dimick et al., 2018), the rich have less support for redistribution than the poor, and the increase in inequality produces higher levels of demand for redistribution in the population. However, since people have a marginal utility of decreasing consumption according to their income, an increase in redistribution is less costly for high-income sectors in terms of wellbeing than for lower strata, which is why the effect of inequality is even greater for the rich than the poor; this limits the differences between both groups in terms of redistributive preferences (Dimick et al., 2016).

Therefore, based on the concepts of social affinity, parochial altruism, and “income-dependent altruism,” the third hypothesis of the study is:

*H3: The greater the inequality of a country, the smaller the differences in redistribution preferences between income levels*.

#### 2.2.2. Economic Development

A second factor at the structural level that the literature has addressed in terms of wellbeing and distributive justice is economic development, commonly measured by the per capita Gross Domestic Product of countries. Among the most classic literature, the link between growth and resource distribution has been marked by the well-known curve proposed by Kuznets (1955): as countries develop, their inequality also increases, to a point where growth begins to return increasingly equitable income distributions.^1^ However, in terms of distributive preferences, economic development has tended to be considered as a control variable (Rudra, 2002; Schmidt-Catran, 2016; Schröder, 2017), with few attempts to establish a direct and explanatory relationship between countries' wealth and their citizens' attitudes toward resource redistribution.^2^

In spite of this, there is a causal mechanism that would not link economic development directly to redistributive preferences but would have a high explanatory power by generating influence on the value configurations of the subjects: the theory of cultural change. According to Inglehart (1977), modernization entails the emergence of post-materialistic values within societies. The increased coverage of basic needs will lead to less economic concerns and more liberal preferences, autonomous and attentive to subsequent needs of personal fulfillment (Inglehart, 2008). This tendency has been linked to the perspectives of solidarity and support for the welfare state (Gelissen, 2000), closely related to the preferences for redistribution.

From this, the following hypotheses are extracted from the research:

*H4a (between): Countries' levels of economic development will be positively related to support for redistribution*.
*H4b (in): Positive changes in countries' economic development will be positively related to support for redistribution*.

Just as the moderating effect of inequality was raised, we have seen how economic development can modify the effect that certain characteristics of people—such as income—have on their own preferences for redistribution. According to Reenock et al. (2007), the emergence of extreme reactions according to socioeconomic strata will occur exclusively in environments characterized by a “regressive socioeconomic distribution,” where accentuated economic development and elementary deficiencies coexist. Coincidentally, Bowles and Gintis (2000) establish that the support of the welfare state tends to be linked to basic moral obligations with others in order to ensure the provision of minimum welfare standards, prioritizing a homo-sociologicus over the homo-economicus of the classical economic conceptions. Therefore, in societies with lower levels of economic development, such as Latin America, where the guarantee and coverage of such basic needs is less assured, self-interest would operate to a lesser extent on the configuration of people's preferences, resulting in fewer differences toward redistribution across income strata (Dion and Birchfield, 2010). Therefore, it is possible to argue that:

*H5: The greater the economic development of a country, the greater the differences in redistribution preferences between income levels*.

**Figure 1:**
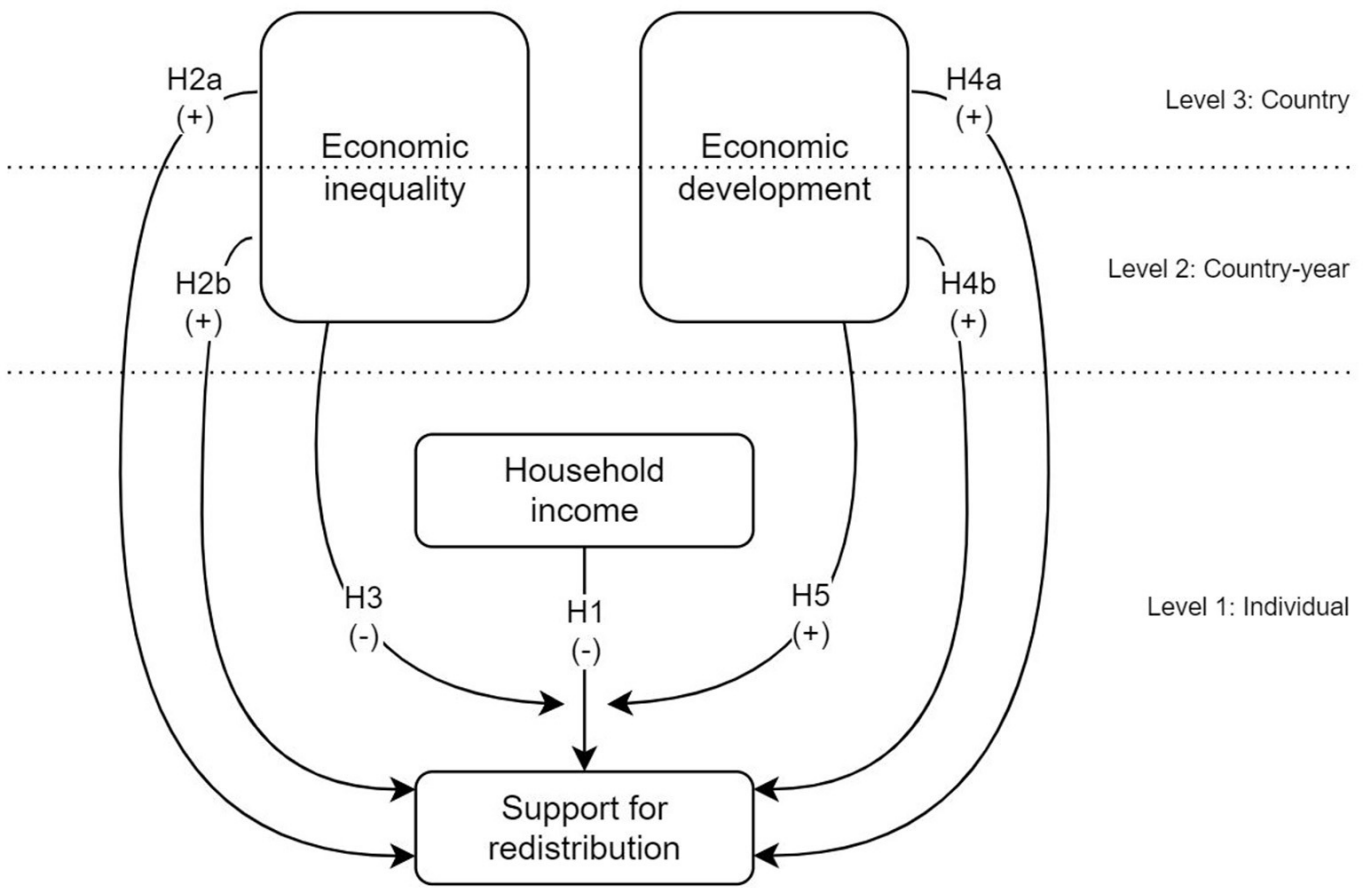
Hypotheses diagram.

Figure 1 summarizes the hypotheses raised. The individual, contextual (country), and temporal (country—year) levels are differentiated since each country has four measurements over time. In addition to the direct effects on redistribution, the dotted line symbolizes the moderating effect of the contextual variables on the relationship between income and support for redistribution.

## 3. Methodology

### 3.1. Data

The data at the individual level come from the Latin American Public Opinion Project (LAPOP) socioeconomic surveys applied to households by the Latin American states themselves. The study includes a stratified sample on three levels, consisting of: 131,787 individuals^3^ (level 1), nested in 97 country units per year (level 2), nested in 17 countries^4^ (level 3).

### 3.2. Variables

#### 3.2.1. Individual Variables

The dependent variable of the study is the individual support for redistribution, measured in the question: “The State [corresponding country] should implement firm policies to reduce income inequality between rich and poor. To what extent do you agree or disagree with this statement?” This variable ranges from 1 (“strongly disagree”) to 7 (“strongly agree”).

The monthly household income is established as the main independent variable. For the 2008 and 2010 waves of LAPOP, the monthly household income is divided into 10 intervals, adjusted to the national currency of each country. However, for 2012, 2014, 2016, and 2018, these intervals are 16. To solve this problem and to be able to measure the effect of the economic location of the subjects with respect to their context on their preferences for redistribution, the 10 categories of the first two waves were maintained, and the income of the last four waves was recoded from 16 to 10 income intervals for each of the country–years. Thus, income is constituted as a continuous variable ranging from 1 (poorest decile) to 10 (richest decile).

The models also consider as controls a series of individual variables that in the literature are considered influential in estimating redistribution preferences (Castillo and Sáez Lozano, 2010). As argued by Brady and Finnigan (2014, p. 21), “consistently, older, female, unmarried, less educated, unemployed, and lower income respondents tend to support more social policies.” That said, it will be controlled by the following variables: (i) gender (female = 0; male = 1); (ii) age, measured in years; (iii) marital status (unmarried = 0; married or cohabiting = 1); (iv) political ideology, in categories “right,” “center,” “left,” and “undeclared”; (v) employment status, in categories “non-working,” “unemployed,” and “employed”; (vi) education, in categories of “primary education complete or less,” “secondary education complete or less,” and “tertiary education incomplete or complete”; and (vii) area of residence (rural = 0; urban = 1). It is also controlled by (viii) trust in the system which, along the same lines as Brandt (2013) and Cichocka et al. (2017), corresponds to the average trust expressed by individuals with respect to various institutions, in this case, six: the Executive, the National Congress, the judicial system, the political parties, the Armed Forces, and the national police; ranging from values of 1 (no trust) to 7 (complete trust).

#### 3.2.2. National Variables

The study considers two national variables: economic inequality and economic development. Economic inequality is measured in the same way that the main studies in the field have done: through the GINI coefficient, which ranges between values of 0 (scenario of complete equality where all individuals have the same income) and 1 (complete inequality where one individual has the entire income). To improve its interpretation, the variable was multiplied by a factor of 100, so that it varies between 0 and 100. In cases where the information was not available for a given year, it was decided to use the information for the year prior to the missing one. Economic development is measured through the annual per capita Gross Domestic Product (GDP) by object of expenditure at constant (2015) prices in thousands of dollars. This indicator is also presented for each country–year unit.

To ensure the robustness of the results, and to control for the heterogeneity not observed by the two national variables included and which could affect people's redistributive conceptions, estimates were also made by integrating the typology of welfare regimes for Latin America developed by Martínez Franzoni (2008).

### 3.3. Hybrid Multilevel Regression Models

To answer the question and the objectives of the research, hybrid multilevel regression models are estimated (Fairbrother, 2014). “This approach uses individual-level data and allows the decomposition of country-level effects into their components across countries (cross-sectional) and within countries (longitudinal)^5^, while simultaneously controlling for individual-level composition effects' (Schmidt-Catran, 2016, p. 3). Equation (1) represents the formula of the models.


(1) $$\begin{array}{r} {\text{y}}_{\text{jti}}=\beta_{0}\left(\text{t}\right)+\beta_{1}{\text{X}}_{\text{jti}}+\gamma_{\text{WE}}\left({\text{Z}}_{\text{jt}}-{\bar{\text{Z}}}_{\text{j}}\right)+\gamma_{\text{BE}}{\bar{\text{Z}}}_{\text{j}}+{\text{v}}_{\text{j}}+{\text{u}}_{\text{jt}}+{\text{e}}_{\text{jti}}\; \end{array}$$

The models envisage the inclusion of three levels, represented in the components of the equation by the sub-indices *j* for countries (level 3), *t* for country–years (level 2), and *i* for individuals (level 1). Thus, individuals are nested in country–years, which are nested in countries.

The *X*_*jti*_ component corresponds to the individual variables, and β_1_ to the coefficients associated with the change in them. The *Z*_*jt*_ component represents a variable at the national level for a given country–year, and ${\bar{Z}}_{j}$ is the average of that variable for the entire period of years, for that country. Thus, γ_*BE*_ accounts for the effect “between” countries, and γ_*WE*_ represents the coefficient associated with the effect of change in that variable “within” a country over time. Likewise, the model controls for unobserved time trends by means of the constant β_0_(*t*). Finally, *v*_*j*_, *u*_*jt*_ and *e*_*jti*_ correspond to the errors at the country, country–year, and individual levels, respectively.

## 4. Results

### 4.1. Descriptive Analysis

Our sample includes 131,787 individuals, nested in 97 country–years (surveys) and 17 Latin American countries, which as shown in Table 1 have, on average, a high level of agreement with the redistribution, materialized by a mean of 5.6 points on a scale ranging from 1 (strongly disagree) to 7 (strongly agree). After the data imputation process previously explained, both the GINI and the GDP per capita are present for all country–years. The GINI coefficient averages 47.7 points, with the lowest at 38.0 points (El Salvador 2018) and the highest at 55.5 points (Honduras 2008). Annual GDP per capita ranges from US$1.679 (Nicaragua 2010) to US$16.037 (Uruguay 2018), with an average of US$7.646 for the 97 country–years.

**Table 1:** Descriptive statistics.

**Statistical**	***n***	**Mean/%**	**SD**	**Min**	**Max**
Support for redistribution	131,787	5.629	1.627	1	7
Household income	131,787	5.058	2.716	1	10
Gender	131,787				
Male		49.9%			
Female		50.1%			
Age	131,787	39.552	15.817	18	112
Family status	131,787				
Married		58.9%			
Not married		41.1%			
Employment	131,787				
No workforce		13.6%			
Unemployed		30.4%			
Employed		56.0%			
Education	131,787				
Primary		29.0%			
Secondary		49.4%			
Tertiary		21.6%			
Political ideology	131,787				
Right		27.0%			
Center		31.7%			
Left		26.2%			
Not declared		15.1%			
System confidence	131,787	3.759	1.347	1	7
Zone	131,787				
Urban		71.0%			
Rural		29,0%			
GINI	97	47.709	4.171	38.000	55.500
GDP per capita	97	7.646	4.122	1.679	16.038

**Figure 2:**
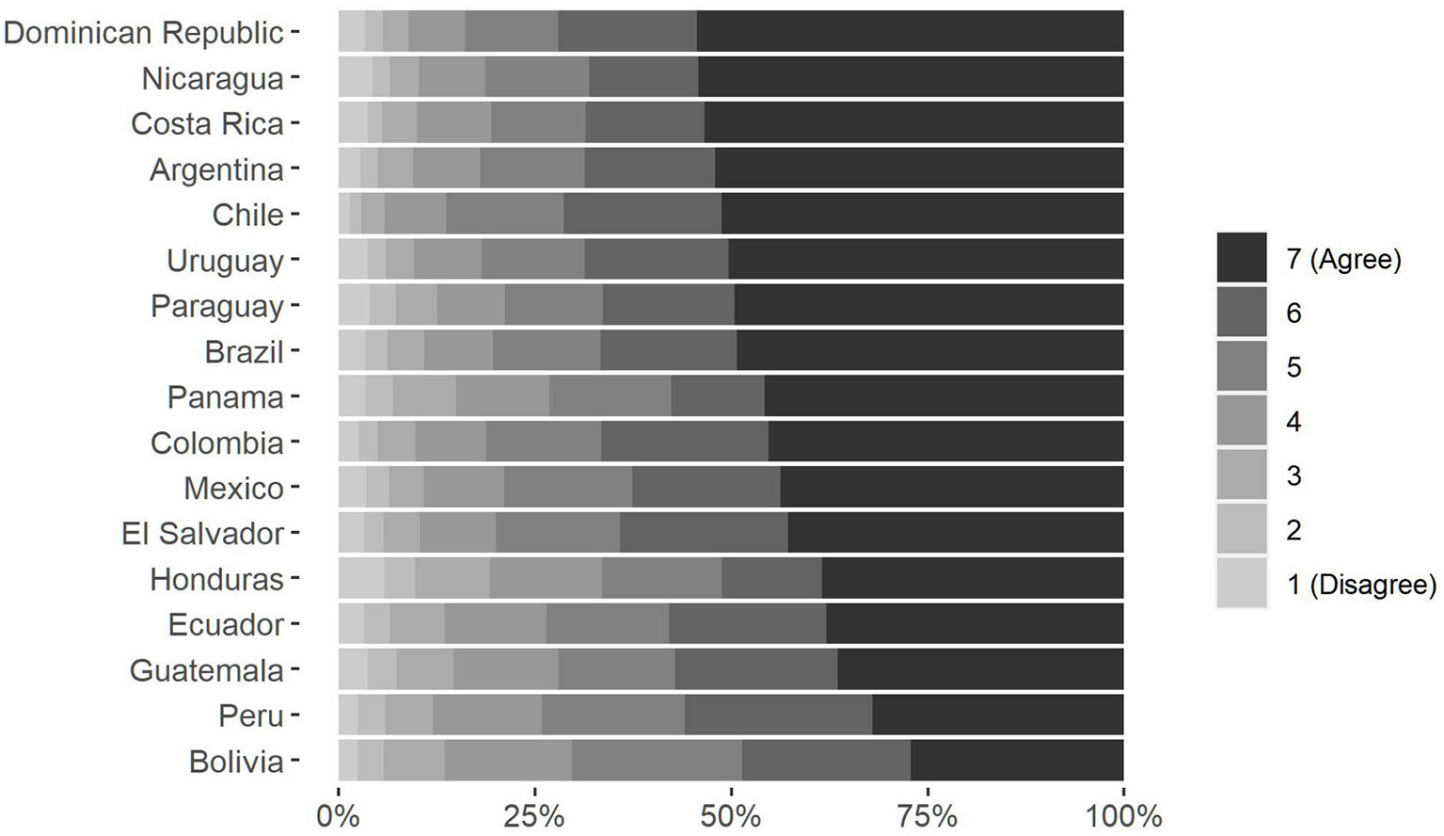
Support for redistribution by countries. Percentage by category.

If we want to describe the region in terms of preferences, there is an essential starting point: most countries express a high demand for redistribution. As can be seen in Figure 2, in all countries more than half of the people fall into categories 6 and 7 of the scale, expressing a high degree of agreement with the redistribution. However, it is also possible to see differences between nations. On the one hand, countries such as Dominican Republic, Nicaragua, Costa Rica, Argentina, Chile, and Uruguay have a very high concentration of individuals who identify with narrowing economic gaps; in these countries, more than 50% of people are in complete agreement with the redistribution of income through the application of strong state policies (category 7). In contrast, in Bolivia and Peru the proportion of people who are completely pro-redistribution does not exceed 33%.

**Figure 3:**
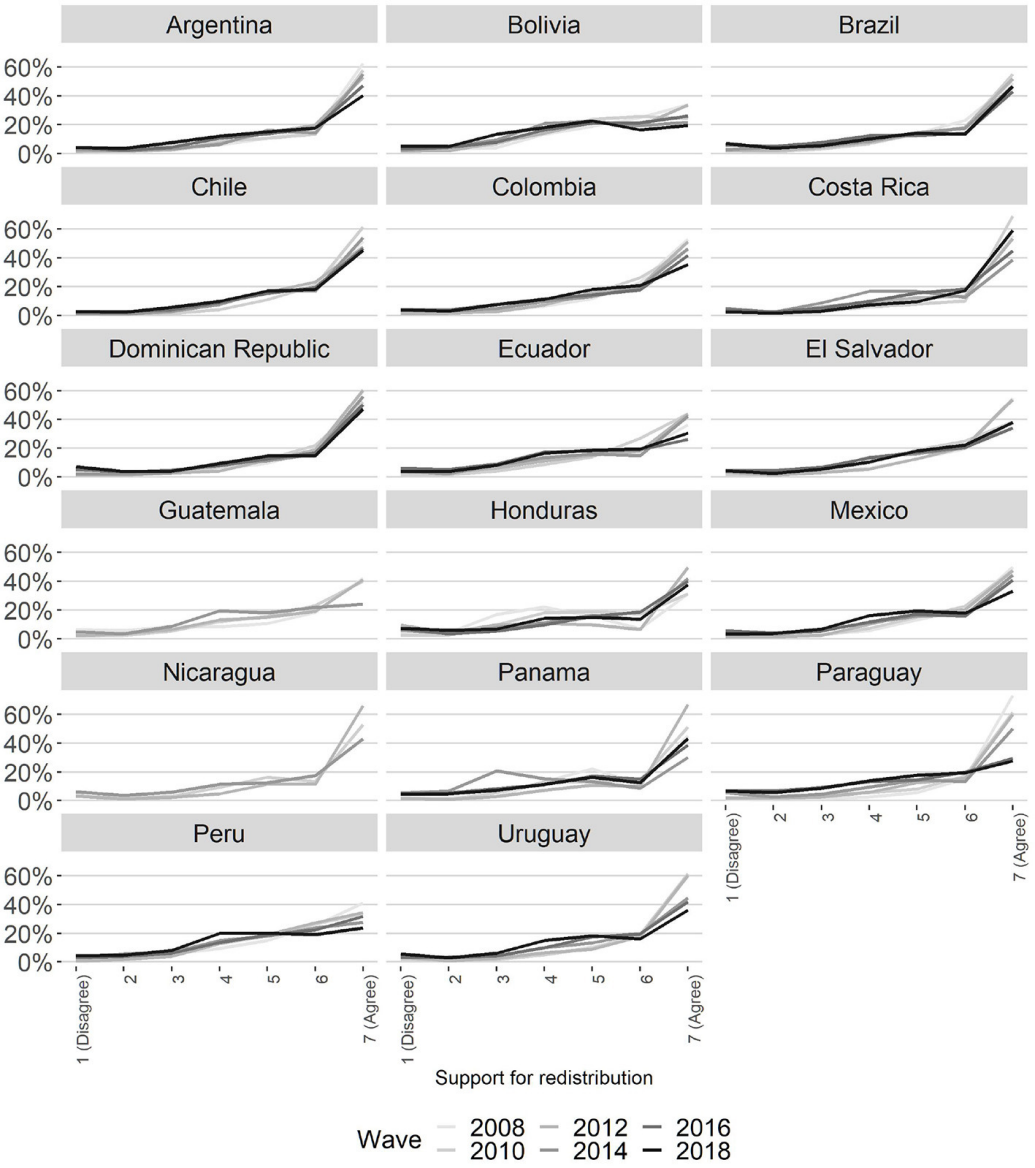
Support for redistribution by country and year. Percentage by category.

Does support for redistribution vary over time within Latin America? How stable are the preferences in this area within each country? As can be seen in Figure 3, the longitudinal behavior of preferences for redistribution is very different from that of the region. While in countries such as Argentina, Chile, Colombia, the Dominican Republic, and Honduras, people's agreement with the redistribution tend to be stable, cases such as Paraguay, Panama, Uruguay, Costa Rica, and Nicaragua show notable variations over time.^6^

Despite the different patterns of stability in redistributive preferences by country, there is a phenomenon that tends to be expressed indistinctly throughout most countries in the region: a decline in levels according to redistribution over time. As shown in Figure 3, most countries express a reduction in the proportion of people absolutely in line with redistribution. The period studied shows a small rise in redistributive preferences in Latin America until 2012 and a subsequent fall until 2018. In concrete terms, the dependent variable expresses an average of 5.76 points for the 2008 sample, 5.85 for 2010, 5.86 for 2012, 5.47 points for 2014, 5.40 points for 2016, and 5.34 points for 2018. Thus, it is possible to state that the demand for redistribution in Latin America is extremely high and in the majority but that it has been declining in longitudinal terms in recent years.

Another central aspect to be evaluated is the association between income and redistributive preferences. As can be seen in Table 2, at the regional level there is no clear pattern between the two phenomena. It could be expected that as people belong to households with higher incomes, their levels of demand for redistribution will be lower, as this state action will imply greater costs than benefits for those segments. However, in Latin America, the lowest averages of support for redistribution are found in the deciles located at both extremes: that is, the richest and poorest deciles. Table 2 shows this, where decile 10 (richest) expresses an average of 5.55 points of support for redistribution, the lowest of all the income intervals, followed by decile 1 (poorest) with an average of 5.58 points. Meanwhile, the lower-middle economic strata (deciles 2, 3, and 4) are those that show greater support for redistribution.

**Table 2:** Average support for redistribution of income deciles, by country.

	**D1**	**D2**	**D3**	**D4**	**D5**	**D6**	**D7**	**D8**	**D9**	**D10**	**Total**
Argentina	5.90	5.97	5.83	5.80	5.87	5.84	5.87	**5.79**	5.85	5.80	5.86
Bolivia*	5.06	5.32	5.35	5.39	5.34	5.24	5.11	5.15	**5.05**	5.07	5.25
Brazil	5.81	5.87	5.77	5.78	5.82	5.63	**5.61**	5.75	5.64	5.62	5.76
Chile*	**5.86**	6.05	6.10	6.07	5.96	5.99	5.99	5.92	5.95	**5.86**	5.99
Colombia	5.70	5.78	5.72	5.89	5.76	5.73	5.81	5.70	5.79	**5.58**	5.76
Costa Rica*	**5.70**	5.86	5.91	5.86	5.88	5.84	5.76	5.84	5.79	5.87	5.84
Dominican Republic*	**5.61**	5.79	5.92	5.96	5.86	5.96	6.04	6.06	6.06	6.04	5.93
Ecuador	5.36	5.41	5.48	5.52	5.55	5.53	5.55	5.39	5.34	**5.12**	5.46
El Salvador*	5.54	**5.52**	5.77	5.75	5.78	5.74	5.68	5.69	5.62	5.63	5.68
Guatemala*	5.32	5.41	5.35	5.45	5.36	**5.30**	5.39	5.45	5.64	5.58	5.40
Honduras*	5.17	5.25	5.37	5.14	5.18	5.15	5.07	**5.04**	5.40	5.48	5.21
Mexico*	5.57	5.71	5.62	**5.55**	5.63	5.65	5.82	5.66	5.67	5.58	5.65
Nicaragua*	5.75	**5.73**	5.92	5.78	5.91	5.83	**5.73**	5.95	5.98	5.75	5.83
Panama	5.53	5.53	5.59	5.60	5.51	5.48	5.52	5.40	**5.31**	5.49	5.51
Paraguay*	**5.38**	5.57	5.57	5.76	5.85	5.86	5.79	5.85	5.74	5.58	5.71
Peru*	**5.20**	5.25	5.37	5.49	5.57	5.48	5.44	5.50	5.55	5.22	5.42
Uruguay*	6.15	5.96	5.85	5.97	5.91	5.76	5.72	5.66	5.65	**5.43**	5.82
Total*	5.58	5.66	5.66	5.66	5.65	5.62	5.62	5.62	5.63	**5.55**	5.63

*In bold, minimum values per country. With an asterisk, countries where there are statistically significant differences between D1 and some other income decile, at a 95% confidence level*.

**Figure 4:**
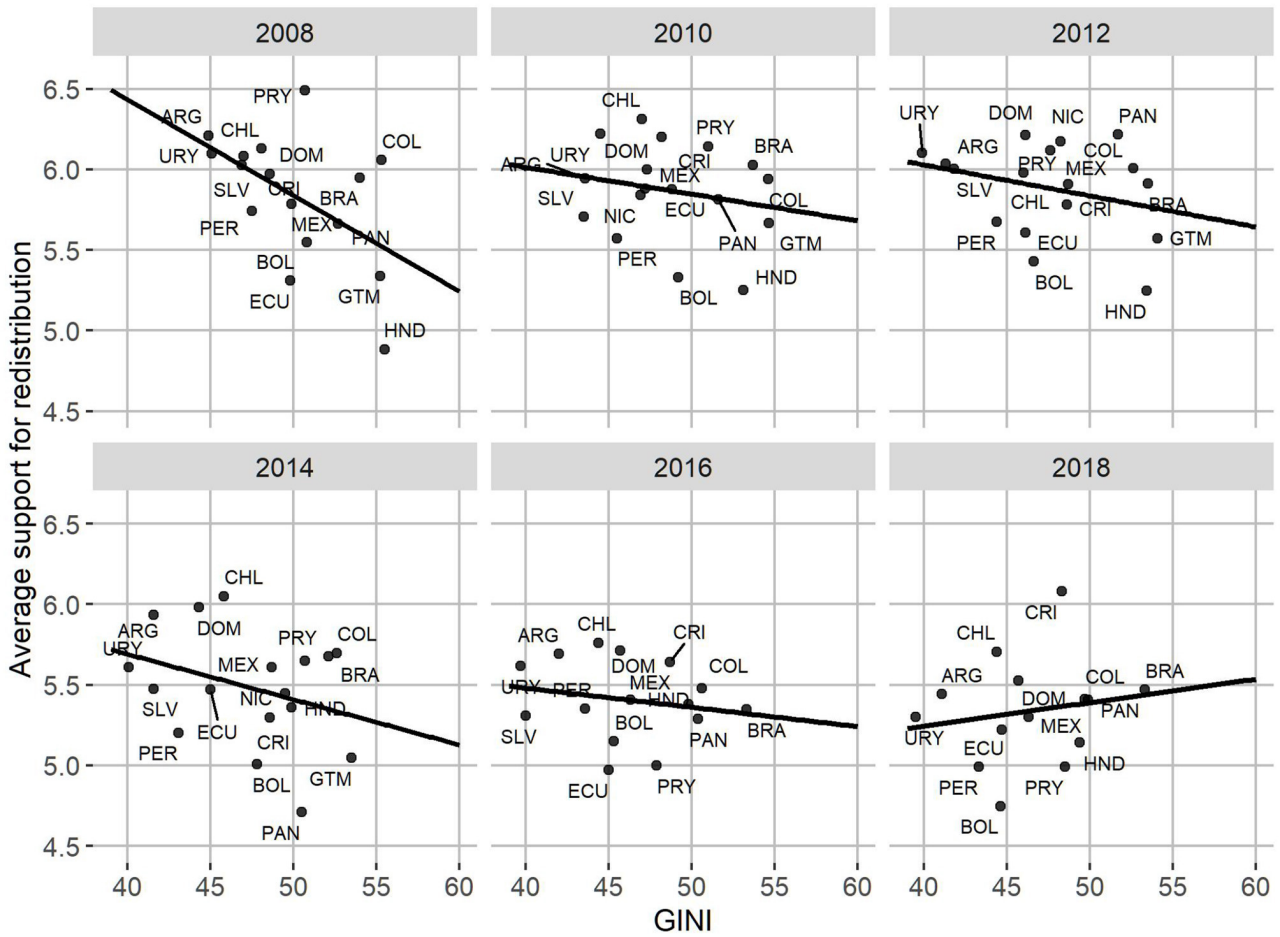
Average support for redistribution and GINI, by country and year.

Regarding the relationship between people's redistributive preferences and the characteristics of the countries in which they live, it is possible to highlight a number of elements. Firstly, Figure 4 shows a predominantly negative association between the GINI coefficient of the countries and the average according to individual redistribution. That is, the more unequal the countries are, the lower their average levels of redistributive preferences tend to be. In 2008 and 2014 the slopes were the most negative in the period under study, while in 2010, 2012, and 2016 they tended to become more moderate. In 2018 it is possible to observe a slightly positive relationship between inequality and redistributive preference, which calls into question the stability of the negative relationship between both factors within the region.

**Figure 5:**
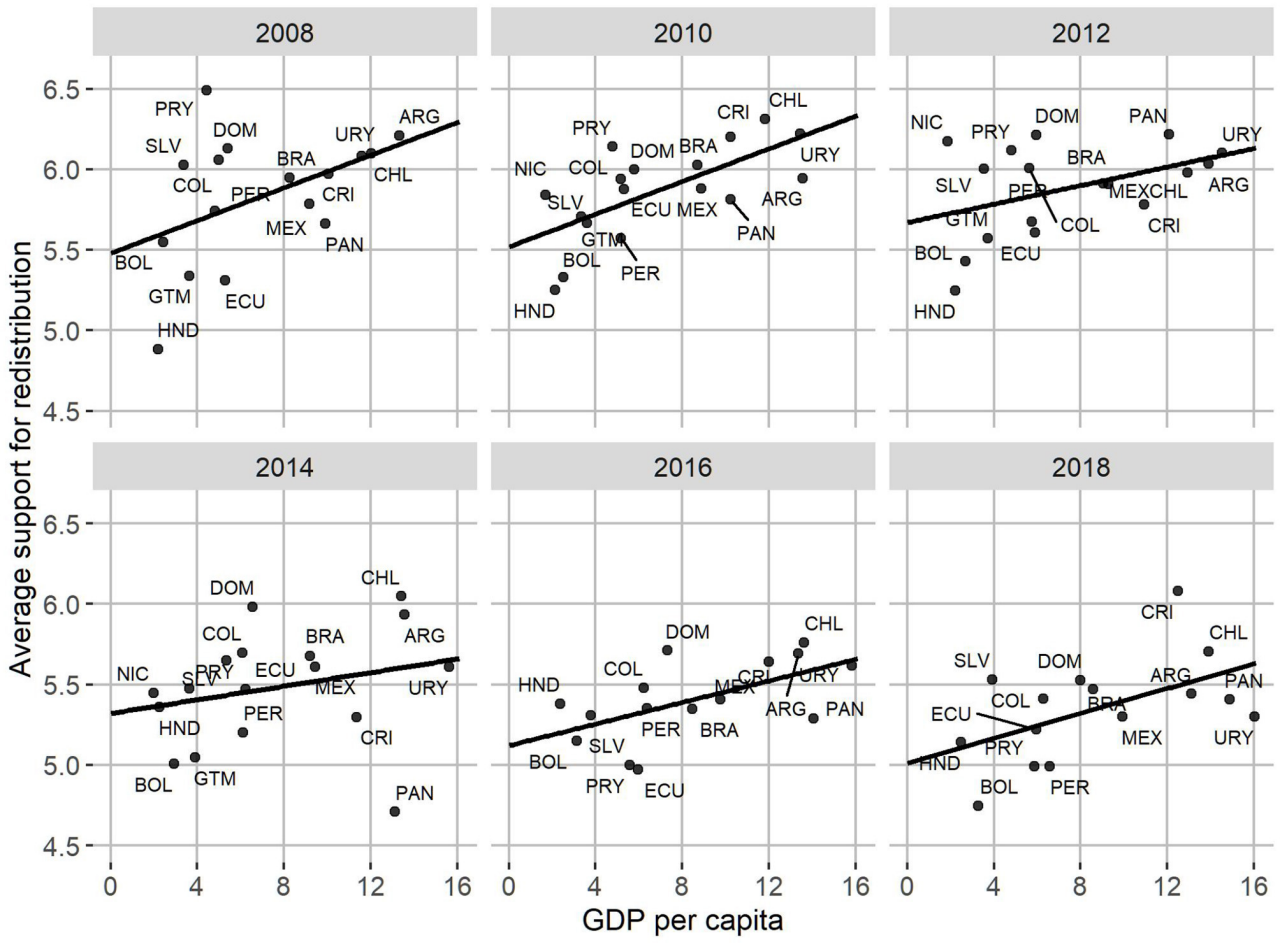
Average support for redistribution and GDP per capita, by country and year.

Secondly, as can be seen in Figure 5, the relationship between individual redistributive preferences and national economic development shows a clearer pattern than previously observed with inequality. Within the region, the richer countries—such as Chile, Uruguay, Brazil, and Argentina—tend to have citizens who are more favorable to reducing inequalities through the application of state policies while within the less economically developed nations—such as Honduras and Bolivia—there is less agreement with the redistribution, despite some exceptions such as El Salvador. Likewise, this positive relationship between economic development and redistributive preferences is constant as it shows little variation over the six time periods studied.

### 4.2. Multilevel Estimation

Given that the objective of this study is to analyze the distribution of redistributive preferences in the 17 countries studied and its variations over time, it is important to begin by pointing out that the dependent variable has an intra-class correlation (ICC) of 0.042 for country–years and 0.015 for countries (ICC according to Hox, 2010, p. 34, Equation 2.16). This means that the variation in the agreement with the redistribution of people within Latin America is about 1.5% due to country membership and 4.2% due to country–year. According to these values, in Latin America, most of the variability in terms of redistributive preferences is related to individual differences and not to the country context or its changes over time. It should also be remembered that the variability of responses on the scale of the dependent variable is restricted (sd = 1,627), which reflects a high consensus in support of redistribution and, therefore, limited space to investigate individual and contextual differences.

Table 3 presents the hybrid multilevel regression models, which estimate the agreement with the redistribution of people based on individual (level 1), country–year (level 2), and country (level 3) variables. Model 1 includes income as an independent and continuous variable, addressing the effect of an increase in one decile of the monthly household income of each country–year. Model 2 also adds control variables at the individual level.

**Table 3:** Hybrid multilevel regression models of individual support for redistribution.

	**Model 1**	**Model 2**	**Model 3**	**Model 4**
**Individual-level variables**				
Income	0.006^***^	0.004^*^	0.004^*^	0.001
	(0.002)	(0.002)	(0.002)	(0.006)
Male		0.025^***^	0.026^***^	0.025^***^
		(0.010)	(0.010)	(0.010)
Age		−0.001^**^	−0.001^**^	−0.001^**^
		(0.000)	(0.000)	(0.000)
Married		0.051^***^	0.051^***^	0.054^***^
		(0.009)	(0.009)	(0.009)
Political ideology				
Center		0.009	0.009	0.007
		(0.012)	(0.012)	(0.012)
Left		0.075^***^	0.075^***^	0.080^***^
		(0.012)	(0.012)	(0.012)
Not declared		0.171^***^	0.171^***^	0.165^***^
		(0.015)	(0.015)	(0.015)
System confidence		0.084^***^	0.084^***^	0.087^***^
		(0.003)	(0.003)	(0.003)
Employment				
Unemployed		−0.000	0.000	0.004
		(0.015)	(0.015)	(0.015)
Employed		0.018	0.018	0.014
		(0.014)	(0.014)	(0.014)
Education				
Secondary		0.096^***^	0.096^***^	0.103^***^
		(0.012)	(0.012)	(0.012)
Tertiary		0.121^***^	0.121^***^	0.135^***^
		(0.015)	(0.015)	(0.015)
Urban		−0.038^***^	−0.038^***^	−0.044^***^
		(0.011)	(0.011)	(0.011)
**Country-level variables**				
GINI[BE]			−0.010	−0.016
			(0.014)	(0.012)
GINI[WE]			−0.008	−0.010
			(0.023)	(0.023)
GDP[BE]			0.032^**^	0.042^***^
			(0.013)	(0.011)
GDP[WE]			−0.020	0.000
			(0.045)	(0.044)
Constant	5.818^***^	5.398^***^	5.646^***^	5.913^***^
	(0.080)	(0.084)	(0.715)	(0.607)
**Time trend**				
2010	0.027	0.001	-0.000	-0.019
2012	0.028	0.014	0.013	-0.010
2014	−0.364^***^	−0.371^***^	−0.368^***^	−0.432^***^
2016	−0.459^***^	−0.447^***^	−0.452^***^	−0.563^***^
2018	−0.513^***^	−0.502^***^	−0.506^***^	−0.657^***^
**Variance components**				
AIC	502135.39	501403.54	501427.34	500999.88
BIC	502233.28	501618.89	501681.85	501293.55
Log Likelihood	−251057.70	−250679.77	−250687.67	−250469.94
N Level 1	131787	131787	131787	131787
N Level 2	97	97	97	97
N Level 3	17	17	17	17
Var: Level 2 (Int)	0.05	0.05	0.05	0.07
Var: Level 2 Income	0.05	0.05	0.03	0.02
Cov: Level 2 (Int) Income	2.50	2.49	2.49	2.48
Var: Level 3 (Int)				0.00
Var: Level 3 Income				−0.01
Cov: Level 3 (Int) Income				0.00
Var: Residual				0.00

*** *p < 0.01*,

** *p < 0.05*,

* *p < 0.1*.

Model 3, on the other hand, integrates all the individual variables included in Model 2 and adds the inequality and economic development of the countries, each broken down into two dimensions. Firstly, the effect “between” countries [BE], represented by the average of the GINI coefficient and GDP per capita per country for the period studied (years 2008, 2010, 2012, 2014, 2016, and 2018); therefore, it is constituted as a level 3 (country) variable. Secondly, the effect of inequality “within” countries [WE] is included, concerning the change in the GINI coefficient and the GDP per capita of each country–year compared to the country average for the period studied of each of those variables. Unlike the “between” countries effect, these variables vary by country for each year, so it is a level 2 (country–year) variable.^7^

Finally, Model 4 works with the same independent variables of Model 3; however, it has the difference that it incorporates random slopes by country–year and country for the effect of household income. Specifically, Model 4 allows the relationship between income deciles and support for redistribution to vary by country–year (level 2) and country (level 3).

#### 4.2.1. Income and Individual Determinants

**Figure 6:**
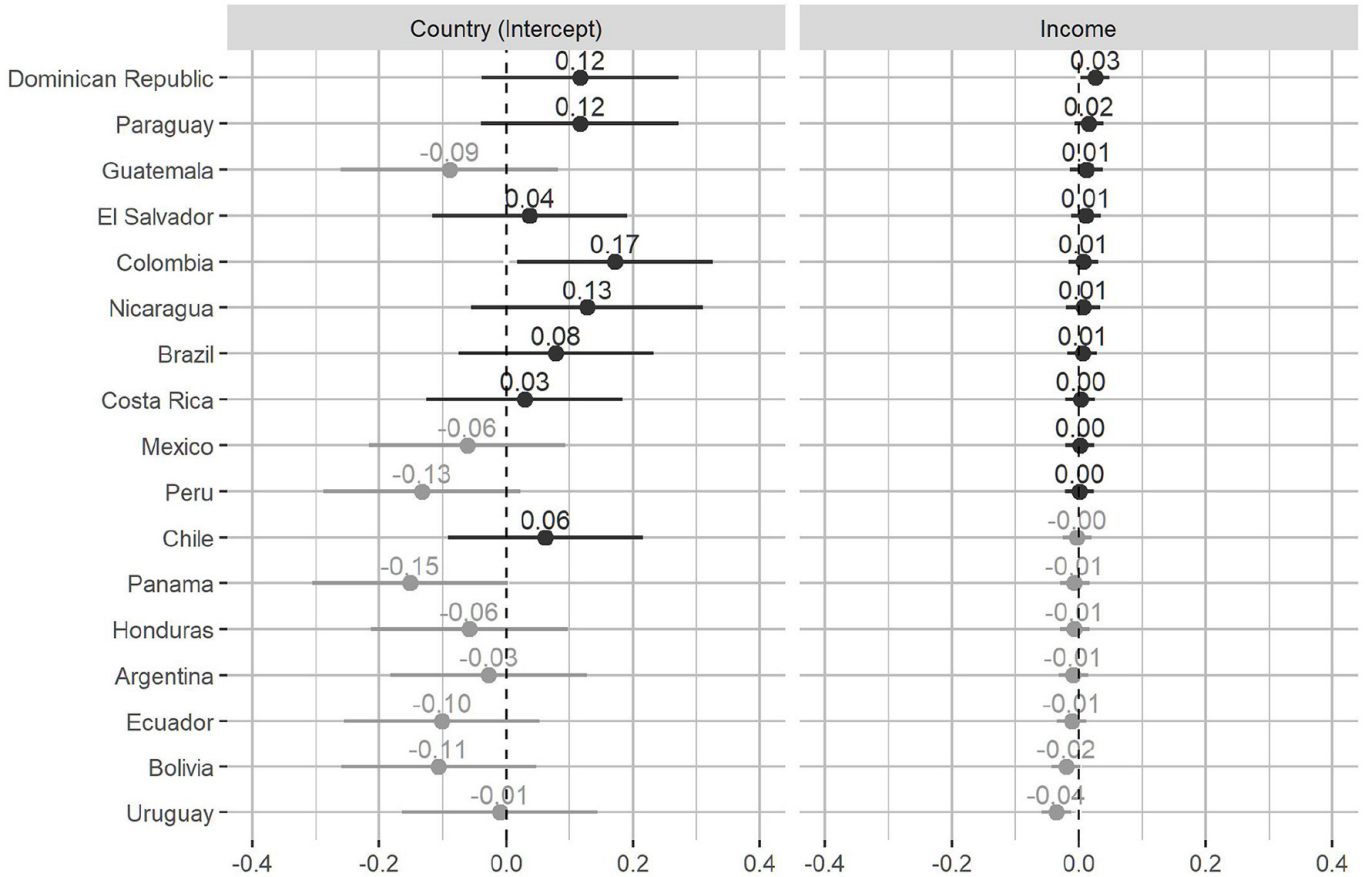
Income random effect on support for redistribution by country: intercept and slope. Points show predicted coefficients; bars represent 95% confidence intervals.

Among the many individual factors that influence redistributive preferences, income is of particular importance in the literature and in this study. As can be seen in Table 3, the effect of income on the support for redistribution within Latin America is statistically significant. Although Model 1 shows an increase of 0.006 points in the scale according to redistribution with an increase of one decile in income, which is significant at 99% confidence, this effect diminishes with the addition of control variables at individual, country–year, and country level in Models 2, 3, and 4. Despite the above, it is highly probable that the relationship between income and redistributive preferences will be different depending on the country in question. Figure 6 shows the random effect of the income variable on the redistributive agreement, by country, obtained from Model 4.^8^

As can be seen, the general trend of weak association between people's economic income and their demand for redistribution tends to remain within the countries of the region. Figure 6, using the income slopes and their confidence intervals, shows that in only 2 of the 17 countries studied in the region does the income decile have a statistically significant effect on the redistributive preferences, even though it is controlled by the other variables: the Dominican Republic where the higher the income, the significantly higher the redistributive preferences, and Uruguay where the higher the income, the significantly lower the redistributive preferences.

The results show that, in contrast to income, other individual factors express a more consistent effect. As can be seen throughout Table 3, the other individual variables behave stably throughout the estimated models, both in terms of magnitude and significance. Within these variables it is relevant to mention education, which at higher levels is consistently associated with greater preferences for redistribution as well as leftist political ideology and confidence in the political system.

#### 4.2.2. Inequality and Economic Development

Concerning the relationship between inequality and redistributive preferences, the estimated models confirm the evidence in the previous descriptive section. Within Latin America, there is a negative association between the economic inequality of nations and people's support for redistribution. This means that as countries become more unequal, people will tend to express a lower degree of support for redistribution. However, this relationship is weak since the coefficients of both the level (GINI[BE]) and change over time (GINI[WE]) of economic inequality do not express statistical significance across the estimated models.

In relation to economic development, it is possible to observe that the levels of economic development between countries (GDP[BE]) show a more evident association with the levels of individual demand for redistribution, showing a positive coefficient of 0.032 points and significant at 95% confidence in Model 3. This number may seem small, but it is still relevant considering the diversity of national economic wealth in the region. On average for the period studied, the poorest country, Nicaragua, presents an average GDP of US$1,837 while Uruguay, the richest, averages US$14,577 per capita. This implies that controlling for all other individual and national factors and taking into account only the effect of country-level economic development, Uruguayans will tend to score 0.408 points higher than Nicaraguans on the redistributive agreement scale, which ranges from 1 to 7. However, this trend is not observed for the change in economic development within countries over time (GDP[WE]) as it manifests a neutral and not statistically significant, effect.

To corroborate the robustness of the results, Model 3 was estimated by controlling for “unobserved heterogeneity” in terms of redistributive preferences, which could be associated with the type of welfare regime in which people live and which could interfere with people's preferences and attitudes toward inequality and redistribution (Schmidt-Catran, 2016). To this end, the typology of welfare regime developed by Martínez Franzoni (2008) for Latin American countries was added to Model 3, which considers three categories: productivist, protectionist, and informal-familialist, the latter being scarcely developed in terms of welfare and social protection within Latin America (Martínez Franzoni, 2008). However, the inclusion of this typology did not generate major modifications in the magnitude of the coefficients and levels of statistical significance expressed in Model 3. Given the small changes involved and appealing to greater parsimony, the models are estimated without the presence of this variable.

#### 4.2.3. Interactions Between Levels

As we have seen so far, income does not show as significant an effect on the levels of preference for the redistribution of individuals as do inequality and, more strongly, the economic development of the countries of the region. Despite the above, it may be perfectly plausible that the economic stratum of people will have an effect in certain scenarios of inequality or economic development. To test these hypotheses, Models 5 and 6 in Table 4 add interactions between levels to assess the possible moderating effect that the GINI coefficient and GDP per capita may have on the relationship between people's income and their individual support for redistribution.

**Table 4:** Hybrid multilevel regression models of individual support for redistribution.

	**Model 5**	**Model 6**
**Individual-level variables**		
Income	−0.115	0.022^*^
	(0.076)	(0.012)
**Country-level variables**		
GINI[BE]	−0.019	−0.017
	(0.012)	(0.012)
GINI[WE]	0.030	−0.010
	(0.025)	(0.023)
GDP[BE]	0.042^***^	0.045^***^
	(0.011)	(0.011)
GDP[WE]	−0.001	−0.071
	(0.044)	(0.048)
**Cross-level interactions**		
Income × GINI[BE]	0.002	
	(0.002)	
Income × GINI[WE]	−0.011^***^	
	(0.002)	
Income × GDP[BE]		-0.003^*^
		(0.001)
Income × GDP[WE]		0.020^***^
		(0.005)
Constant	6.022^***^	5.902^***^
	(0.608)	(0.601)
**Individual-level controls**	Yes	Yes
**Year fixed effects**	Yes	Yes
**Variance components**		
AIC	501003.39	501008.10
BIC	501316.64	501321.34
Log Likelihood	−250469.70	−250472.05
N Level 1	131787	131787
N Level 2	97	97
N Level 3	17	17
Var: Level 2 (Int)	0.06	0.06
Var: Level 2 Income	0.00	0.00
Cov: Level 2 (Int) Income	−0.00	−0.00
Var: Level 3 (Int)	0.02	0.02
Var: Level 3 Income	0.00	0.00
Cov: Level 3 (Int) Income	0.00	0.00
Var: Residual	2.48	2.48

*Cross-level interactions*.

*** *p < 0.01*,

** *p < 0.05*,

* *p < 0.1*.

As shown in Model 5, the levels of inequality between countries (GINI[BE]) do not express significant effects; however, the change in inequality over time (GINI[WE]) does moderate the effect of belonging to a richer income decile. In concrete terms, for each point of GINI coefficient that countries increase compared to their average for the period studied, the effect of income on redistribution becomes 0.011 points more negative, at 99% statistical confidence. In substantive terms, this implies that our Hypothesis H3 is rejected: in Latin America when inequality within the country increases, the differences between economic strata in terms of redistributive preferences are greater, causing the richer sectors to be associated with lower and lower levels of support for redistribution.

As with inequality, the level of economic development between countries (GDP[BE]) does not generate significant differences, but the change in economic development over time (GDP[WE]) does have implications for redistributive preferences by economic stratum. As Model 6 expresses, as a country's GDP per capita increases by one thousand dollars, the effect associated with belonging to a higher decile of income in redistribution is increased by 0.020 points, a significant change at the 99% confidence level. Contrary to Hypothesis H5, within the region, the rise in countries' economic development increases the differences between income strata in their demand for redistribution.

**Figure 7:**
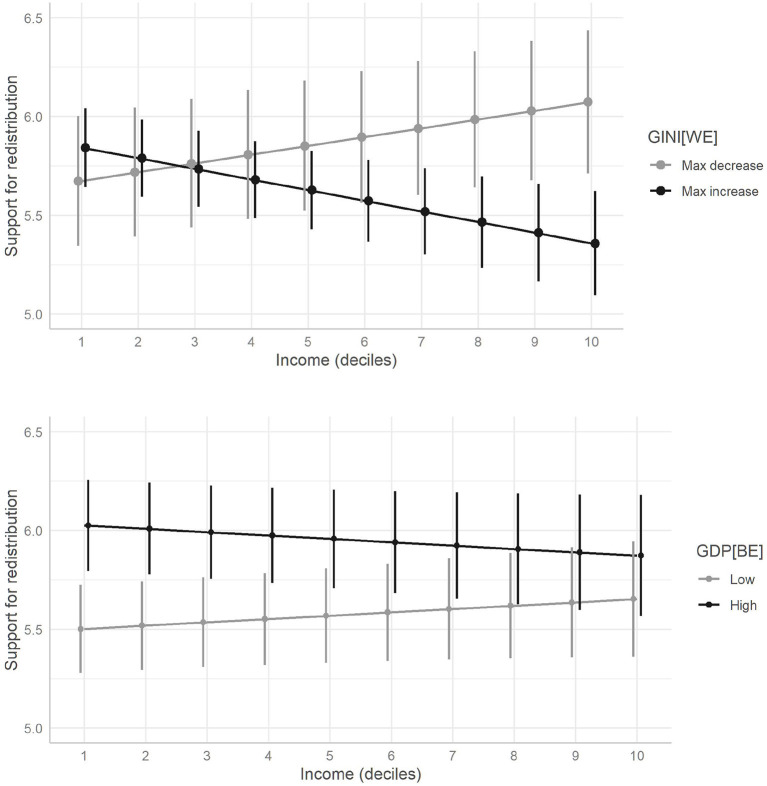
Predicted values of support for redistribution as a function of income deciles with different inequality changes (GINI[WE]) and economic development levels (GDP[BE]). Dots show predicted values; bars represent 95% confidence intervals.

The results of the analysis show the capacity of inequality and economic development in countries to alter the relationship between people's income and their support for redistribution. However, the question remains of which economic strata are particularly susceptible to seeing their preferences modified in terms of the different scenarios of inequality and economic development within Latin America? Figure 7 responds to this question by drawing on the results of Models 5 and 6, expressing the predicted values according to redistribution for each income decile, in terms of different changes in inequality (GINI[WE]) and levels of economic development (GDP[BE]).^9^

As can be seen, inequality and economic development have implications for different economic positions in terms of redistributive preferences. As shown in Figure 7, inequality has implications only for the richest (10th) income decile as this is the only economic stratum that shows statistically significant differences between the scenarios of maximum decrease and maximum increase in inequality observed in the countries and periods studied.^10^ Furthermore, the change in inequality can modify the slope of the predicted values of the redistributive agreement vs. income, this relationship being negative when inequality increases over time and vice versa.

On the contrary, economic development implies differences exclusively in the poorest segments. From decile 1 to decile 4 of income, there are statistically significant differences in the agreement with the redistribution predicted for scenarios of low and high GDP per capita^11^ between countries (GDP[BE]), while in the remainder of the richer strata it is not possible to find differences.

## 5. Discussion

This study aimed to characterize redistributive preferences and their changes in an unequal and developing context, such as Latin America. In descriptive terms, the first thing that should be emphasized is the high degree of support for redistribution within the region. In Latin America, the population is mostly in agreement with the reduction of inequalities via the state in all the countries studied. This is in alignment with other studies, which position Latin America—along with the Middle East—as the region with the highest levels of support for redistribution in the world (Dion and Birchfield, 2010). However, this support for redistribution shows changes over time. Overall, there is evidence of a sustained decline in the levels of people's redistributive preferences, particularly from 2014 onwards. In this regard, the average support for redistribution tends to be more stable in countries such as Honduras, Chile, and Mexico and acquires greater variation in Paraguay and Panama.

This research has a series of implications for the study of redistributive preferences, considering their limited development in the region and the absence of studies from a longitudinal perspective. Firstly, the results question the hegemonic approaches to preferences for redistribution, based on self-interest as well as their universalist pretensions. Unlike what has tended to be stated in other contexts, such as Europe (Schmidt-Catran, 2016), in Latin America it is possible to observe an absence of a relationship between people's income and their agreement with the application of public policies to reduce inequalities. Within the region, the economic stratum to which individuals belong is not associated with changes in redistributive preferences. As mentioned, controlling for other relevant individual and national factors, belonging to a higher decile of family income is associated with a lower support for redistribution only in Uruguay, while in the Dominican Republic it is the opposite, and in the remainder of the countries income is not associated with differences in individuals' support for redistribution. Contrary to what is commonly postulated by classical economic theories, in the region people's redistributive preferences are not guided by a direct cost-benefit relationship based on the objective economic position of individuals. This lack of relationship may be due to the low implications of the economic stratum in the configuration of preferences or to the lack of knowledge that people have regarding their objective position as has been seen in other research in developed countries (Engelhardt and Wagener, 2018).

The absence of a relationship between income and support for redistribution in the region allows us to confirm, through the use of cross-sectional data, the specificity of unequal and developing contexts in terms of attitudes toward inequality. Our findings are consistent with those previously presented by Dion and Birchfield (2010), who also revealed that in countries with low levels of economic development or high degrees of inequality, people's income does not satisfactorily explain their support for redistribution.

In agreement with other contemporary authors (Amable et al., 2019), this research questions the historical hegemony of theories based on self-interest for the understanding of redistributive preferences. As evidenced by all the estimates developed, people's educational level is inversely related to the demand for redistribution: the higher the educational level, the greater the support for redistribution. Considering the lower exposure to risk faced by the more educated, in a logic of self-interest one would expect their support for redistribution to be lower, but the opposite is true. Likewise, people's working condition, which is fundamental in the relationship people might have with welfare policies, is not a determining element in their support for redistribution, as Berens (2015a) previously observed in Latin America.

Regarding contextual variables, the influence of the economic development of the country within the region stands out as higher levels are associated with higher levels of redistribution. This element also expresses differences with the research that has been done on the subject in other regions. Authors such as Schmidt-Catran (2016) explain how in Europe citizenship of richer countries is associated with lower levels of demand for redistribution. The positive effect of economic development on preferences for income redistribution in Latin America can be explained by its link to reductions in poverty, which the region has experienced in recent years (Birdsall et al., 2011; Dayton-Johnson, 2015) and which Wietzke (2016) endorses as having an important role in supporting redistribution for developing countries. The particularity of the effect that economic development has specifically for the support for redistribution of the poorest segments, seen in the results of this study, reaffirms this explanation.

Unlike economic development, inequality manifests a less evident and even contrary influence. Our results show that in Latin America, higher levels of inequality in countries are associated with a decrease in the degree of agreement with the redistribution of people. Also, we observe that the wealthy segments are particularly susceptible to changes in the inequality of countries in terms of redistributive preferences, as stated by the theory of “income-based altruism” (Dimick et al., 2016, 2018), but in a direction contrary to this as higher levels of inequality are capable of triggering lower levels of support for redistribution in the higher-income group. Within the region, inequality is even capable of decreasing the altruism of higher-income individuals.

These phenomena could be explained by the divergence, empirically proven in many contexts, between objective and subjective inequality (Castillo, 2010; Sachweh and Olafsdottir, 2010; Mijs, 2019). More than changes in the actual levels of inequality, what could generate modifications in the support for redistribution would be the perceptions, beliefs, and judgments toward inequality (Janmaat, 2013) that are predominant in each of the countries. According to Cramer and Kaufman (2011), the differences between income strata in terms of dissatisfaction with the existing inequality in Latin America are not enhanced when the levels of objective inequality increase either. Given this, the highly unequal Latin American context can be understood as an interpretative framework that is strongly rooted in people's preferences, constant, and independent of progress or setbacks in terms of distribution.

## 6. Conclusion

This research has evidenced various findings in the configuration and change of redistributive preferences in Latin America. Firstly, it has been found that the application of public policies to limit existing inequalities tends to be widely supported by the Latin American population, but that this majority agreement has tended to diminish in recent years. Likewise, it has been shown that people's income, a traditional determinant in the configuration of redistributive preferences, does not generate major differences in the demand for redistribution within the region. On the contrary, educational level and ideological factors, such as political ideology and confidence in the political system, are much more influential variables.

In addition, while most of the support for redistribution within Latin America is explained by individual factors, it is possible to detect implications for factors in the national context. In countries with greater economic development, people's redistributive preferences tend to be greater, particularly among the poorest sectors who identify with significantly higher levels of support for redistribution. In contrast, when countries increase their economic inequality over time, membership in wealthier deciles is associated with even lower levels of demand for redistribution. All in all evidence at individual and country level suggest a series of limitations of classical rational and self-interest theories to understand and explain the dynamics of redistributive preferences in Latin America.

Research on redistributive preferences and attitudes toward inequality has tended to be carried out mainly in developed countries while paradoxically regions such as Latin America are those with the greatest problems in terms of distribution. This empirical shortage entails a series of problems and limitations that all new research in the field must deal with and to which this study is not exempt. The main problem refers to the difficulty in obtaining quality longitudinal data series for developing regions. For this reason, this research only examines the effects of inequality and economic development, given that these are the determinants at the country level with the best quality information, knowing that there are so many others—government social spending, labor informality, immigration rates to name a few—that the literature has seen as capable of influencing attitudes toward welfare policies.

Another limitation of the study is the time gap that could exist between the structural conditions to which people are exposed and their attitudes in terms of distribution^12^ as well as the problems associated with the operationalization of the household income variable.^13^ However, the testing of the median-voter theorem and the self-interest approach, in its essence, assumes the use of this variable as the purest representation of the cost-benefit ratio that such approaches have tended to defend as a supposed determinant in the articulation of attitudes toward redistribution.

Finally, the longitudinal approach of this study sheds some light on aspects that are not perceptible with cross-sectional analysis. In this line, the downward trend of redistributive preferences shows a highly relevant research aspect to be explored further. That, in one of the most unequal regions on the planet, people year after year are less in support for redistribution is without a doubt a momentous phenomenon in matters of public policy, political economy, and economic sociology. Contextual studies and studies of socio-historical trends are some of the varied research strategies that could be employed to respond to these types of questions—tremendously interesting considering the wide range of challenges that the region presents in terms of distribution.

From the findings revealed by this research, several questions arise that require further study to be correctly understood. First, consider not only “how much” but “who” redistributes. The high rates of institutional corruption within the region, and the importance that confidence in the political system has shown in explaining variations in the degrees of support for redistributive action, make this a necessary approach to the problem in question, especially in Latin America, a region marked by the fragility of its institutions (Portes and Smith, 2010) and various internal differences in political, cultural, and economic terms, factors that can impact on people's political perceptions (Stevens, 2016). While trust in the state and its institutions is established as one of the most influential determinants of how much people support the redistribution of resources, the promotion of skepticism toward the system could become, paradoxically, a highly effective instrument by Latin American political elites.

The importance of confidence in the political system reveals the strength of environmental perceptions in forming welfare policy judgments. For this reason, the interaction of theoretical approaches such as self-interest and ideological attitudes would be a particularly appropriate avenue to pursue to analyze with greater specificity the factors that make the relationship between the economic stratum of belonging and the sustained agreement toward redistribution more complex. In addition, the influence of cultural values and ideological positions is an element that could further sophisticate the relationship between economic stratum and agreement with the application of public policies to diminish inequalities.

## Data Availability Statement

Publicly available datasets were analyzed in this study. This data can be found at: https://github.com/justicia-distributiva/preferences-redistribution-LA.

## Author Contributions

GF: literature review, data manipulation, estimation of statistical models, and discussion. J-CC: introduction, discussion, conclusions, and GitHub. Both authors contributed to the article and approved the submitted version.

## Funding

The research for this article was supported by the National Research and Development Agency (ANID) under FONDECYT Grant number 1210847 and the Centre for Social Conflict and Cohesion Studies (ANID/Fondap-15130009).

## Conflict of Interest

The authors declare that the research was conducted in the absence of any commercial or financial relationships that could be construed as a potential conflict of interest.

## Publisher's Note

All claims expressed in this article are solely those of the authors and do not necessarily represent those of their affiliated organizations, or those of the publisher, the editors and the reviewers. Any product that may be evaluated in this article, or claim that may be made by its manufacturer, is not guaranteed or endorsed by the publisher.

## Footnotes

^1^Although originally formulated for industrialized nations, this theorem has been applied to a wide range of contexts (Alvaredo, 2010; Atkinson et al., 2011; Williamson, 2015).

^2^Among those few, Finseraas (2009), within a sample of 22 European nations, establishes that those more developed have on average less support for redistribution, but its effect does not manage to be statistically significant.

^3^Observations that have valid values for all the variables of interest at an individual level, except for the variable of political ideology, where cases that do not declare it are included to avoid a greater bias of the sample.

^4^Cuba, Puerto Rico, and Venezuela are excluded, given their scarcity of economic information at the country level.

^5^Also known as “between” and “within” country effects.

^6^To more clearly observe the variations in theaverages according to the redistribution by country, through the different years of measurement, review the Appendix.

^7^The effects “between” and “within” tend to be abbreviated as “BE” and “WE,” for their expression in English: “between effect” and “within effect”.

^8^To confirm this statement, Model 2 was estimated with different measures of household income: continuous (as presented in Model 2), categorical (with income deciles as differentiated categories), and quadratic. These estimates can be found in the Appendix.

^9^Control variables adjusted to the mean or most frequent category at the individual level.

^10^Max decrease = −3.97; max increase = 4.93.

^11^Low = −1.65; high = 13.98.

^12^Along these lines, Schröder (2017) shows how levels of real inequality are capable of predicting a subsequent tolerance to income inequality, in a period of three to four years. Although in no case does it constitute a threat to the veracity and robustness of the results, in the future it should be an element to be considered for research designs on the matter.

^13^For more information see Feres (1997).
