# Supplementary material for: Preferences for Income Redistribution in Unequal Contexts: Changes in Latin America Between 2008 and 2018
Source: Front Sociol. 2022 May 4;7:806458. doi: 10.3389/fsoc.2022.806458 (PMC9114507; doi:10.3389/fsoc.2022.806458)
Supplement: Supplementary file 1 [file Data_Sheet_1.PDF]

7 APPENDIX 1

Table 5. Sample: Observations by country and year.

	2008	2010	2012	2014	2016	2018	Total
Argentina	1028	1041	1027	868	1123	1260	6347
Bolivia	2487	2408	2473	2356	1407	1440	12571
Brazil	1234	2142	1369	1344	1305	1264	8658
Chile	1288	1620	1301	1108	1410	1377	8104
Colombia	1213	1322	1197	1355	1275	1302	7664
Costa Rica	1252	1083	1032	1104	1258	1320	7049
Dominican Republic	1165	1246	1244	1296	1147	1278	7376
Ecuador	2674	2728	1329	1273	1238	1281	10523
El Salvador	1427	1451	1193	1272	1322	1209	7874
Guatemala	1059	1146	1088	1212			
Honduras	1233	1458	1299	1369	1187	1083	7629
Mexico	1288	1336	1232	1130	1317	1331	7634
Nicaragua		1258	1447	1359			
Panama	1355	1434	1314	1374	1306	1338	8121
Paraguay	988	1073	1274	1082	1049	1294	6760
Peru	1337	1343	1291	1138	2299	1320	8728
Uruguay	1328	1370	1314	1378	1353	1437	8180
Total	22356	25459	22424	22018	19996	19534	131787

8 APPENDIX 2

Table 6. Within and between-country distribution of dependent and independent variables.

Country	Support for redistribution			GINI		GDP	
	Mean	SD total	SD years	Mean	SD years	Mean	SD years
Argentina	5.86	1.55	0.25	42.42	1.38	13.47	0.25
Bolivia	5.25	1.55	0.27	47.38	2.15	2.83	0.32
Brazil	5.76	1.62	0.25	53.32	0.60	8.71	0.33
Chile	5.99	1.35	0.20	45.77	1.07	12.88	0.89
Colombia	5.76	1.53	0.25	52.57	1.99	5.72	0.50
Costa Rica	5.84	1.62	0.30	48.50	0.18	11.18	0.88
Dominican Republic	5.93	1.56	0.24	46.20	1.22	6.50	0.90
Ecuador	5.46	1.65	0.29	46.57	2.00	5.77	0.35
El Salvador	5.68	1.58	0.27	41.97	2.78	3.59	0.21
Guatemala	5.40	1.69	0.24	54.36	0.64	3.71	0.12
Honduras	5.21	1.85	0.17	51.85	2.28	2.27	0.12
Mexico	5.65	1.62	0.23	47.85	1.35	9.41	0.35
Nicaragua	5.83	1.65	0.30	48.22	1.04	1.84	0.12
Panama	5.51	1.73	0.47	51.13	0.95	12.38	1.85
Paraguay	5.71	1.70	0.57	49.40	1.43	5.13	0.50
Peru	5.42	1.56	0.27	44.57	1.54	5.80	0.63
Uruguay	5.82	1.60	0.34	41.47	2.37	14.58	1.45
Total	5.63	1.63		47.71	4.17	7.65	4.12

9 APPENDIX 3

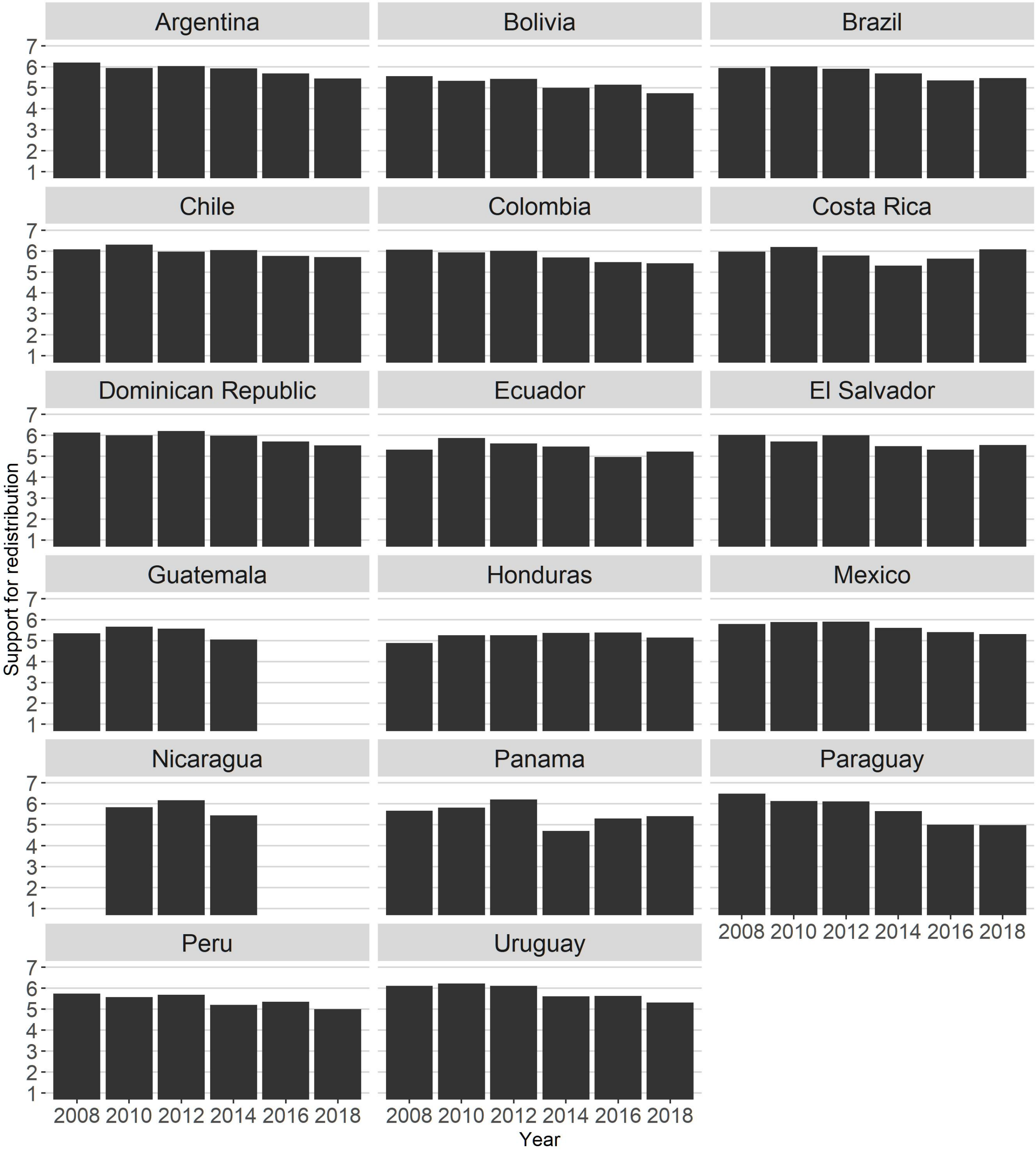

Figure 8. Average support for redistribution, by country and year.

10 APPENDIX 4

Table 7. Hybrid multilevel regression models of individual support for redistribution. Continuous, categorical and quadratic income measures.

	Model 1	Model 2	Model 3
Income	0.004^{*}		0.026^{***}
	(0.002)		(0.007)
Income^2			-0.002^{***}
			(0.001)
Income_Decile2		0.044^{**}	
		(0.019)	
Income_Decile3		0.054^{***}	
		(0.019)	
Income_Decile4		0.058^{***}	
		(0.019)	
Income_Decile5		0.076^{***}	
		(0.020)	
Income_Decile6		0.047^{**}	
		(0.021)	
Income_Decile7		0.056^{***}	
		(0.021)	
Income_Decile8		0.061^{***}	
		(0.022)	
Income_Decile9		0.102^{***}	
		(0.023)	
Income_Decile10		0.015	
		(0.023)	
Constant	5.398^{***}	5.362^{***}	5.352^{***}
	(0.084)	(0.085)	(0.085)
Individual-level controls	Yes	Yes	Yes
Year fixed effects	Yes	Yes	Yes
AIC	501403.54	501438.84	501408.03
BIC	501618.89	501732.51	501633.17
Log Likelihood	−250679.77	−250689.42	−250681.01
N Level 1	131787	131787	131787
N Level 2	97	97	97
N Level 3	17	17	17
Var: Level 2 (Int)	0.05	0.05	0.05
Var: Level 3 (Int)	0.05	0.05	0.05
Var: Residual	2.49	2.49	2.49

$^{***}p < 0.01$, $^{**}p < 0.05$, $^{*}p < 0.1$
